# Bleeding risk in patients with venous thromboembolic events treated with new oral anticoagulants

**DOI:** 10.1007/s11239-020-02319-w

**Published:** 2020-11-02

**Authors:** Niklas Wallvik, Henrik Renlund, Anders Själander

**Affiliations:** 1 Department of Public Health and Clinical Medicine, Umeå University, 981 87 Umeå, Sweden; 2 Uppsala Clinical Research Center, Uppsala University, Uppsala, Sweden

**Keywords:** New oral anticoagulants, Venous thromboembolism, Major bleeding, Risk factors

## Abstract

New oral anticoagulants (NOACs) is the preferred treatment in secondary prophylaxis of venous thromboembolic events (VTE). The aim of this study was to investigate possible risk factors associated with major bleeding in VTE-patients treated with NOACs. In this retrospective register-based study we screened the Swedish anticoagulation register *Auricula* (during 2012.01.01–2017.12.31) to find patients and used other national registers for outcomes. Primary endpoint was major bleeding defined as bleeding leading to hospital care. Multivariate Cox-regression analysis was used to reveal risk factors. 18 219 patients with NOAC due to VTE were included. 85.6% had their first VTE, mean age was 69.4 years and median follow-up time was 183 days. The most common NOAC was rivaroxaban (54.8%), followed by apixaban (42.0%), dabigatran (3.2%) and edoxaban (0.1%). The rate of major bleeding was 6.62 (95% CI 6.19–7.06) per 100 treatment years in all patients and 11.27 (CI 9.96–12.57) in patients above 80 years of age. Statistically independent risk factors associated with major bleeding were age (normalized HR 1.38, CI 1.27–1.50), earlier major bleeding (HR 1.58, Cl 1.09–2.30), COPD (HR 1.28, CI 1.04–1.60) and previous stroke (HR 1.28, Cl 1.03–1.58) or transient ischemic attack (TIA) (HR 1.33, Cl 1.01–1.76). Prior warfarin treatment was protective (HR 0.67, CI 0.58–0.78). This real world cohort shows a high bleeding rate especially among the elderly and in patients with previous major bleeding, COPD and previous stroke or TIA. This should be considered when deciding on treatment duration and NOAC dose in these patients.

**Electronic supplementary material:**

The online version of this article (10.1007/s11239-020-02319-w) contains supplementary material, which is available to authorized users.

## Highlights

- Register study to examine risk factors for major bleeding on NOAC-treatment due to VTE.
- High age, history of major bleeding, COPD and previous stroke or TIA increase the risk of major bleeding.
- Previous anticoagulation with warfarin confers lower bleeding risk.
- The rate of major bleeding is especially high among elderly (11.27 per 100 treatment years).

## Introduction

The incidence of first VTE in the general population is about 0.1% per year [1, 2]. Total mortality associated with the diagnosis of acute pulmonary embolism is 8–17% in three months follow-up time and more than doubles after discharge from hospital [3]. In 1960, a medical breakthrough was made when vitamin K-antagonists (VKAs) was introduced as oral anticoagulant treatment of VTE [4]. A systematic review of patients with VTE on vitamin K antagonist treatment found a risk of recurrence of 3.5% (0.5% lethal) and a risk of major bleeding of 1.6–2.1% (0.2% lethal) during 3–6 months [5]. After 3 months, the risk of major bleeding is estimated to be 0.8% per patient year or 2,6 times more than without anticoagulant treatment [6]. A meta-analysis of available randomized controlled trials (RCTs) comparing NOACs with warfarin in treatment of acute VTE reported an equivalent efficacy but significantly lower risk of major bleeding, 1.1% vs 1.7% (RR 0.6 95% CI 0.4–0.9) during mainly a treatment period of 6 months. Irrespective of treatment group, there was a negligible risk of lethal pulmonary embolism and no difference in mortality (2.4% vs 2.4%) [7]. Risk factors for major bleeding on low molecular weight heparin (LMH) and VKAs in patients with VTE are age > 65, earlier bleeding, cancer, kidney failure, thrombocytopenia, low time in therapeutic range (TTR), surgery, alcoholic abuse and fall [6]. In a register study from a Swedish cohort, independent risk factors of major bleeding on VKA-treatment in VTE-patients were identified as age, cardiac failure, chronic obstructive pulmonary disease (COPD), alcoholic abuse, anemia, hypertension and a history of major bleeding [8]. Risk factors for major bleeding in patients on NOACs as well as warfarin has been extensively studied in atrial fibrillation, but not in patients with VTE. Anemia or history of major bleeding, age > 75, vascular disease, abnormal renal function, abnormal liver function, excessive alcohol, antiplatelet agents or non-steroidal anti-inflammatory drugs are described as risk factors in patients with NOACs due to atrial fibrillation [9]. In addition, certain metabolically interactive substances seem to be of importance [10]. The bleeding risk with NOACs in atrial fibrillation has also been studied specifically in the elderly (mean age 81.6) and estimated to 4.4% for major bleeding and 5.7% for clinically relevant non-major bleeding at 12 month follow-up [11]. Risk factors for major bleeding in patients with NOAC treatment due to VTE might distinguish from atrial fibrillation. VTE-patients of course has another underlying disease, they are five-ten years younger and even though they share characteristics like occurrence of smoking and obesity they also diverse in comorbidities like presence of cancer [1, 12, 13]. The aim of this study was to investigate possible risk factors associated with major bleeding in VTE-patients treated with NOACs.

## Material and methods

The study was approved by the ethical research committee in Umeå, Dnr 2015/145-31. Data were collected from the Swedish national quality register for oral anticoagulation (Auricula), the Swedish National Patient Register (NPR) and The Cause of Death Register (CDR). According to the latest published yearly report *Auricula* has over 150 000 active patients, the coverage ratio is estimated to be about 50% with no apparent selection bias, NOAC represents about half of the treatment years and the VTE indication constitutes 18% of all patients with oral anticoagulation [14]. The *NPR* contains diagnoses defined as ICD-10 codes on hospital admissions and outpatient clinics for patients with a Swedish personal identity number. About 99% has a valid personal number and current absence of primary diagnosis at hospital admission or in outpatient clinics is only 0.9% and 3.2% respectively [15]. The *CDR* consists of all identified deaths in Sweden with a personal identity number and information includes age, sex, date and cause of death.

In this study *Auricula* was used to find patients on treatment with NOAC due to a physician reported VTE between 2012.01.01 and 2017.12.31. Individual patients could only be included once, therefore, in patients registered several times, only the first treatment period was included. Using personal identity number included patients where then identified in the *NPR* and the *CDR* to find characteristics at baseline and endpoints (see Appendix A for ICD-10 definitions). Both primary and secondary diagnosis were used to maximize baseline characteristics and to avoid missing possible endpoints. Characteristics at baseline data were collected and compared for bleeders and non-bleeders as well as for different age groups (< 60, 60–80 and > 80 years = elderly) using non-parametric tests (Chi-square test was used for categorical data and Kruskal Wallis test for age between groups). Primary endpoint was major bleeding defined as bleeding leading to inpatient or specialized outpatient hospital care. Patients contributed with follow-up time for major bleeding until that endpoint occurred or until the end of 2017. Follow-up also ended in the event of death or if treatment stopped or changed in terms of dosage or substance. Major bleedings were subcategorized if ICD-coded as gastrointestinal or intracranial. Any bleeding that led to hospital care was termed as other bleeding. R Core Team (Version 2020; R Foundation for Statistical Computing, Vienna, Austria) was used for data analysis. Incidence rates were calculated for bleeding end-points (major bleeding, gastrointestinal bleeding, intracranial bleeding and other bleeding) in all patients and for the different age groups. Cumulative risk of bleeding end-points were presented with Kaplan Meier survival curves. Both univariate and multivariate Cox regression tests were used to screen possible risk or protective factors for major bleeding. 95% confidence intervals were used.

## Results

In total 18 219 patients with NOAC-treated deep vein thrombosis (DVT) or pulmonary embolism (PE) were included in the study. The mean age was 69.3 years and 52.4% were males. Among included patients 38.8% had acute DVT, 9.1% had recurrent DVT, 40.5% had acute PE, 5.3% recurrent PE and 6.3% DVT or PE not classified as acute or recurrent. The majority had a VTE for the first time (85.6%). The overall distribution of NOAC substances were rivaroxaban 54.8%, apixaban 42.0%, dabigatran 3.2% and edoxaban 0.1%. Low dose NOAC (i.e. rivaroxaban 10 mg × 1, apixaban 2.5 mg × 2, dabigatran 110 mg × 2 or edoxaban 30 mg × 1) was used in 13.2% of the patients. The median follow-up time was 183 days (IQR 92-360). Characteristics at baseline when NOAC-treatment was started are presented for all patients in Table 1 (with separate columns for bleeders and non-bleeders) and Online Resource 1 (with separate columns for different age groups).

**Table 1:** Characteristics for all patients and major bleeders at baseline

	Total, n = 18 219 n (%)	Bleeders, n = 938 n (%)	Non-bleeders, n = 17 281 n (%)	*p*-value
Age, *median* (IQR)	69.4 (56.8–78.5)	74.2 (65.1—82)	69.1 (56.4—78.3)	< 0.01
Sex (male)	9 544 (52.4)	461 (49.1)	9 083 (52.6)	0.045
First DVT/LE^a^	15 587 (85.6)	767 (81.8)	14 820 (85.8)	< 0.01
Prior warfarin treatment	5 682 (31.2)	359 (38.3)	5 323 (30.8)	< 0.01
Hypertension	1 606 (8.8)	120 (12.8)	1 486 (8.6)	< 0.01
Myocardial infarction	1 119 (6.1)	78 (8.3)	1 041 (6)	< 0.01
PCI^b^	148 (0.8)	11 (1.2)	137 (0.79)	0.28
Atrial fibrillation	1 243 (6.8)	118 (12.6)	1 125 (6.5)	< 0.01
Heart Failure	892 (4.9)	79 (8.4)	813 (4.7)	< 0.01
TIA	1 532 (8.4)	57 (6.1)	510 (3)	< 0.01
Stroke	1 134 (6.2)	101 (10.8)	1033 (6)	< 0.01
Vascular disease	1 587 (8.7)	114 (12.2)	1 473 (8.5)	< 0.01
Diabetes	963 (5.3)	52 (5.5)	911 (5.3)	0.77
COPD^c^	1 491 (8.2)	112 (11.9)	1 379 (8)	< 0.01
Dementia	252 (1.4)	11 (1.2)	241 (1.4)	0.67
Anemia	748 (4.1)	61 (6.5)	687 (4)	< 0.01
History of major bleeding	3 428 (18.8)	351 (37.4)	3 077 (17.8)	< 0.01
Gastrointestinal	888 (4.9)	112 (11.9)	776 (4.5)	< 0.01
Intracranial	452 (2.5)	52 (5.5)	400 (2.3)	< 0.01
Previous other bleeding	2 416 (13.3)	235 (25.1)	2 181 (12.6)	< 0.01
Renal failure	343 (1.9)	28 (3)	315 (1.8)	0,02
Excessive alcohol use	558 (3.1)	40 (4.3)	518 (3)	0.04
Fall	4 298 (23.6)	275 (29.3)	4 023 (23.3)	< 0.01
Liver disease	168 (0.92)	13 (1.4)	155 (0.9)	0.18
Cancer	2 145 (11.8)	149 (15.9)	1 996 (11.6)	< 0.01
Cancer in GI-tract^d^	387 (2.1)	27 (2.9)	360 (2.1)	0.13

^a^DVT/PE not classified as first or secondary excluded

^b^Percutanous coronary intervention

^c^Chronic obstructive pulmonary disease

^d^Gastrointestinal tract

**Fig. 1:**
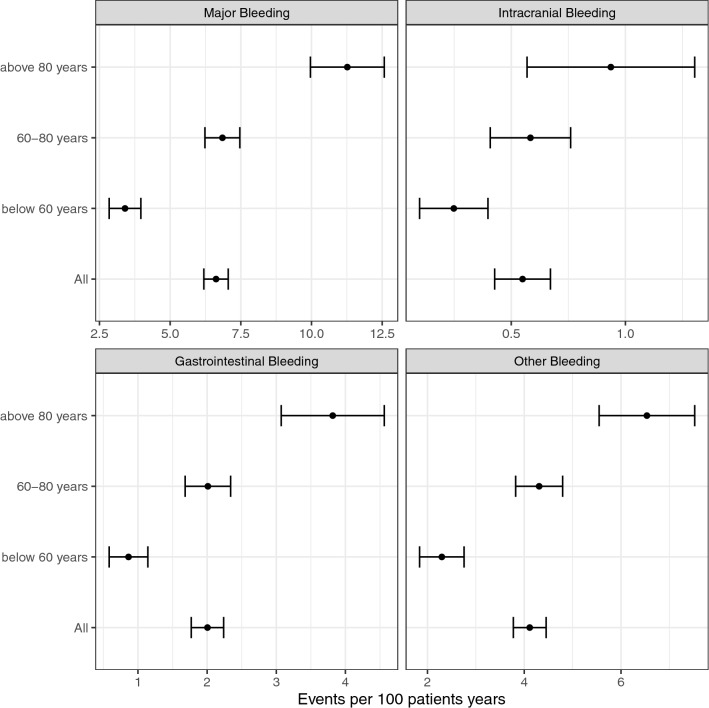
Bleeding rates per 100 patient years for all patients and in different age groups (< 60 years, 60–80 years and > 80 years). Rates for major bleeding, gastrointestinal, intracranial and other bleeding are presented in separate boxes with means and 95% confidence intervals

**Fig. 2:**
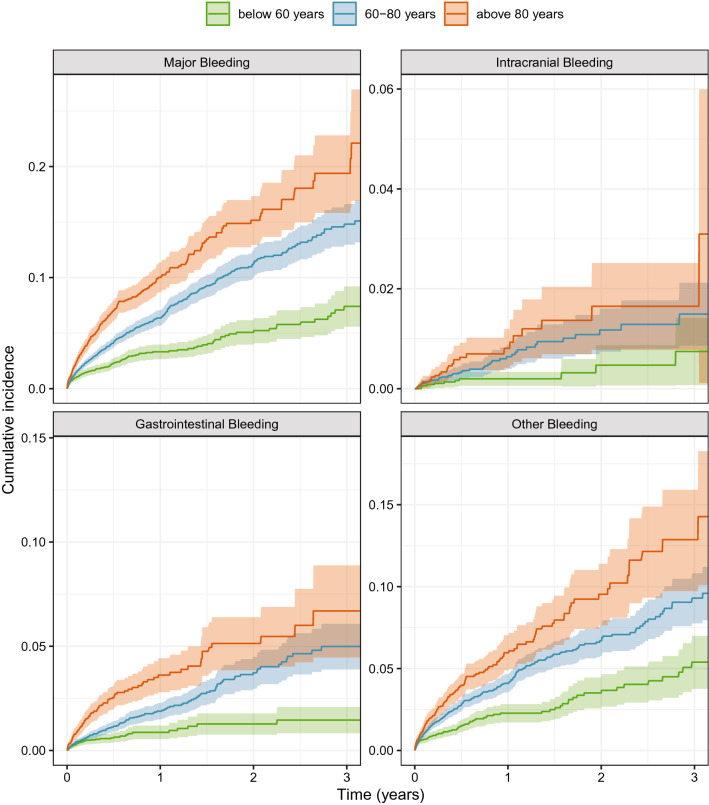
Age stratified cumulative risk of major bleeding. Cumulative risk of bleeding (y-axis) depending on years from treatment start (x-axis) in different age groups (red:  > 80 years, blue: 60–80 years and green:  < 60 years). Separate boxes for major bleeding, gastrointestinal bleeding, intracranial bleeding and other bleeding

Among the 18,219 patients 938 had a major bleeding and the rate of major bleeding was 6.62 (95% CI 6.19–7.06) per 100 treatment years. Intracranial bleeding was found in 81 patients, 293 patients had gastrointestinal and 590 patients other bleedings, with corresponding bleeding rates of 0.55 (CI 0.43–0.67), 2.00 (CI 1.77–2.24) and 4.11 (CI 3.77–4.45) respectively. Major bleeding rate for patients under 60 years was 3.40 (CI 2.84–3.97), between 60 and 80 years 6.85 (CI 6.23–7.47) and over 80 years 11.27 (CI 9.96–12.57) (Fig. 1). The cumulative risk of major bleeding was significantly increased with higher age (Fig. 2). A similar pattern was observed for gastrointestinal bleeding and other bleeding but the risk of intracranial bleeding was not significantly different between the age groups.

**Fig. 3:**
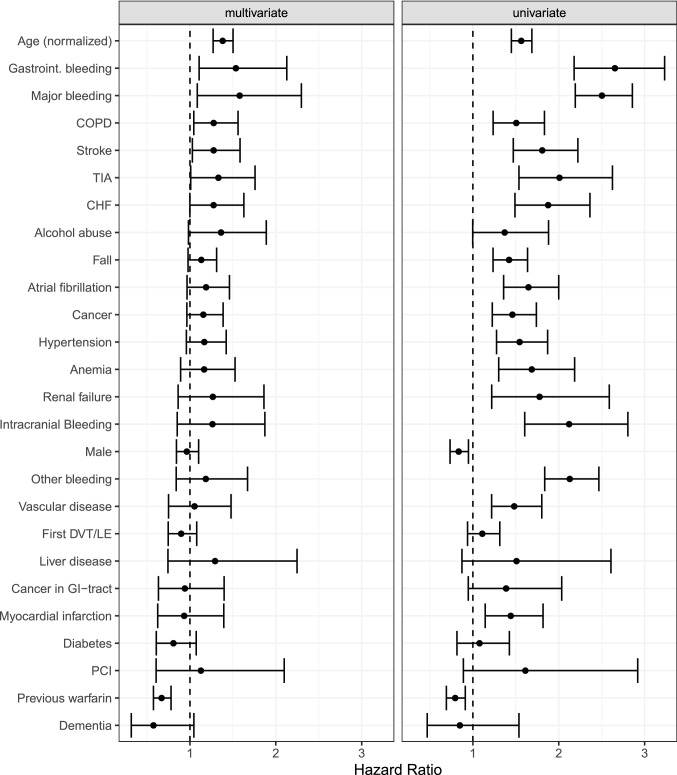
Univariate and multivariate Cox regression analysis with major bleeding endpoint. Hazard ratios for different variables with 95% confidence intervals. Numbers are presented in Online Resource 2

Results of univariate and multivariate Cox regression are presented in Fig. 3. Exact numbers are presented in Online Resource 2. Univariate Cox regression analysis indicate that female sex, hypertension, myocardial infarction, atrial fibrillation, heart failure, vascular disease, anemia, renal failure, fall tendency and cancer were associated with a higher risk of major bleeding but these risk factors were not confirmed in multivariate analysis. Statistically significant risk factors associated with major bleeding after multivariate Cox regression analysis were age (HR 1.38, CI 1.27–1.50), previous stroke (HR 1.28, Cl 1.03–1.58) or transient ischemic attack (TIA) (HR 1.33, Cl 1.01–1.76), COPD (HR 1.28, CI 1.04–1.60) and earlier major bleeding (HR 1.58, Cl 1.09–2.30). Prior warfarin treatment was a protective factor (HR 0.67, CI 0.58–0.78).

## Discussion

In this real-world cohort of patients with VTE on treatment with NOACs, the overall rate of major bleeding was 6.62 per 100 treatment years, with almost doubled bleeding rate [11, 11] among the elderly (> 80 years), driven by an increase in both gastrointestinal and other non-intracranial bleeds. Compared to the two major clinical trials EINSTEIN-VTE and AMPLIFY the major bleeding rate is about 5% higher in absolute numbers [16, 17]. This is probably due to differences in cohort characteristics, study design and definitions of events. A possible explanation for the overall high bleeding risk in our study could be a broader definition of major bleeding which here is dependent on the physicians’ clinical diagnosis rather than strict criteria of hemoglobin drop or the need of blood transfusion. False positive bleeding rates is also likely when based on administrative ICD-codes without a complementary chart review [18, 19].

A similar study to this using administrative ICD-10 codes for estimating bleeding rates, but with real life data on warfarin with well-regulated TTR instead of NOACs in treatment for venous thromboembolism found a lower risk of major bleeding and reported a major bleeding rate of 2.36 per 100 treatment years [8]. This might be due to a slightly older population in our study even though we in general report lower comorbidity. Our cohort might also be more prone to bleeding since the proportion of previous major bleeding was considerably higher (18.8% compared to 7.1%). Historic major bleeding has been identified as an important risk factor for major bleeding in other studies [8, 9, 20]. Most of our patients also had a venous thrombosis for the first time (85.6% compared to 44.3%) and had therefore probably to a lesser extent been exposed to anticoagulation before. Both studies report a protective effect of prior warfarin treatment and this might add to the explanation of the higher bleeding rate in this study. Furthermore, rivaroxaban together with apixaban dominated this study and recent observation studies on oral anticoagulation in atrial fibrillation reports that rivaroxaban is not protective regarding major bleeding [21] and might even be harmful compared to warfarin [22].

Cox regression analysis revealed age, previous stroke or TIA, COPD and historic major bleeding as independent risk factors associated with major bleeding. Prior warfarin treatment was a protective factor which probably reflect the fact that these patients are a selected group that tolerates anticoagulation well and therefore are less prone to bleed. These findings differentiate slightly from the results of a prospective study in patients with anticoagulation as stroke prophylaxis due to atrial fibrillation [9], but since that study was not a pure study on NOACs the comparability is obscured. Identified risk factors there which were not confirmed in our study were vascular disease, abnormal renal function, abnormal liver function and excessive alcohol use. However, findings of age and history of major bleeding as risk factors were the same. Our results are also to some extent different from the similar observation study on warfarin treatment due to VTE [8]. There, atrial fibrillation actually was a protective factor and hypertension, cardiac failure, alcohol abuse and anemia were identified as risk factors. This was not found in our study with patients on NOACs due to VTE. However, the common findings of age, COPD and history of major bleeding strengthens the reliability of our results. Some questions still remain. How is it that COPD increases the bleeding risk? If the increased risk of bleeding in patients with previous stroke or TIA is explained by concurrent antiplatelet therapies why is it not the same in myocardial infarction? Possibly, COPD or previous stroke could be markers for more frail patients, which could account for some of the increased bleeding risk.

## Conclusion

A high rate of major bleeding causing in-hospital care was found especially in the elderly and in patients with previous stroke or TIA, COPD or history of major bleeding. In these patient groups, the risk of major bleeding should be of concern for the physician deciding on dose and treatment duration of NOACs as secondary prophylaxis for VTE. Extended anticoagulation with low dose NOAC in clinical equipoise situations is supported by two RCTs [23, 24]. In the oldest patients, a reduced NOAC dose could be considered more often due to their higher bleeding rate, although further studies are needed to determinate the efficacy of low dose NOACs in acute VTE in the elderly.

## Limitations

This is a retrospective register-based study with all limitations that comes with it and bias cannot be ruled out. Data depends on physicians subjective diagnostic approach and the validity for bleeding end-points based on ICD-codes are questionable. Prevalence of underlying parameters such as hypertension and alcohol abuse could be underestimated since information from the primary health care is lacking in the NPR. The possibility of interactions effects such as concurrent antiplatelet also remains undetectable.

### Electronic supplementary material

Below is the link to the electronic supplementary material.

Supplementary file1 (DOCX 17 kb)

Supplementary file2 (DOCX 17 kb)

## Data Availability

Raw data are not attached.

## Appendix A: ICD-10 codes

**Table:**

Venous thromboembolism	I26, I636, I676, I80-82
Hypertension	I10-15
Myocardial infarction	I21-22, I24, I252
Percutanous coronary intervention (PCI)	FNG02, FNG05, Z955
Atrial fibrillation	I48
Heart Failure	I50, I110, I130, I132
Valve malfunction	I05-08, I34-36, I39, Q22-24, T82
Stroke	I63-64, I69
TIA	G450-453, G458, G459
Vascular disease	I21-22, I252, I70-71
Diabetes	E10-14
Chronic obstructive pulmonary disease	J40-70
Dementia	F00-03
Anemia	D50, D510, D513, D518, D519, D52, D53, D55, D560-D562, D568-D572, D588, D589, D59-64
Major bleeding	I60-I62, S064-066, R04, K250, K252, K254, K256, K260, K262, K2264, K266, K270, K272, K274, K276, K280, K282, K282, K286, K920-922, K290, I850, I983, K625, D629, R589
Gastrointesinal bleeding	I850, I983, K250, K252, K254, K256, K260, K262, K264, K266, K270, K272, K274, K276, K280, K282, K284, K286, K625, K920-922
Intracranial bleeding	I60-I62, S064-066
Other bleeding	D500, D508-509, D629, H356, H922, N02, N938-939, R04, R310
Renal failure	I120, I131-132. N17-19, DR016, DR024, KAS00, KAS10, KAS20
Excessive alcohol use	F10, K70, T51, Y90, Y91, E244, G312, G621, G721, I426, K292, K860, O354, Z714
Fall(s)	W00-19
Liver disease	K70-77, JJC, JJB
Cancer	C1-C9
Cancer in GI-tract	C15-C26
Intracerebral bleeding	I60-I62, S064-066

## Code availability

R Core Team (Version 2020; R Foundation for Statistical Computing, Vienna, Austria) was used for data analysis.
