# Supplementary material for: Bleeding risk in patients with venous thromboembolic events treated with new oral anticoagulants
Source: J Thromb Thrombolysis. 2020 Nov 2;52(1):315–23. doi: 10.1007/s11239-020-02319-w (PMC8282556; doi:10.1007/s11239-020-02319-w)
Supplement: Supplementary file 1 — Supplementary file1 (DOCX 17 kb) [file 11239_2020_2319_MOESM1_ESM.docx]

# Bleeding risk in patients with venous thromboembolic events treated with new oral anticoagulants

*Authors*: Niklas Wallvik^1,3^, Henrik Renlund^2^, Anders Själander^1^

^1^ Umeå University, Department of Public Health and Clinical medicine, 981 87, Umeå, Sweden

^2^ Uppsala Clinical Research Center, Uppsala University, Uppsala, Sweden

^3^ Corresponding author. E-mail: niklas.wallvik@gmail.com

**Online Resource 1. Characteristics for included patients presented in different age groups at baseline.**

|  | <60 years, n=5 352 | 60-80 years, n=8 964 | >80 years, n=3 903 | *p*-value |
| --- | --- | --- | --- | --- |
|  | n (%) | n (%) | n (%) |  |
| Age, *median* (IQR) | 48.9 (40.2-55.0) | 70.7 (66.2-75.0) | 85.4 (82.6-88.7) | N/A |
| Sex (male) | 3 190 (59.6) | 4 963 (55.4) | 1 391 (35.6) | <0.01 |
| First DVT/LE^1^ | 4 590 (85.8) | 7 604 (84.8) | 3 393 (86.9) | 0.67 |
| Prior warfarin treatment | 1 511 (28.2) | 2 821 (31.5) | 1 350 (34.6) | <0.01 |
| Hypertension | 173 (3.2) | 896 (10.0) | 537 (13.8) | <0.01 |
| Myocardial infarction | 87 (1.6) | 558 (6.2) | 474 (12.1) | <0.01 |
| PCI^2^ | 6 (0.1) | 91 (1.0) | 51 (1.3) | <0.01 |
| Atrial fibrillation | 66 (1.2) | 620 (6.9) | 557 (14.3) | <0.01 |
| Heart Failure | 60 (1.1) | 394 (4.4) | 438 (11.2) | <0.01 |
| TIA | 32 (0.6) | 262 (2.9) | 273 (7.0) | <0.01 |
| Stroke | 85 (1.6) | 535 (6.0) | 514 (13.2) | <0.01 |
| Vascular disease | 131 (2.4) | 816 (9.1) | 640 (16.4) | <0.01 |
| Diabetes | 149 (2.8) | 525 (5.9) | 289 (7.4) | <0.01 |
| COPD^3^ | 319 (6.0) | 808 (9.0) | 364 (9.3) | <0.01 |
| Dementia | 5 (0.1) | 108 (1.2) | 139 (3.6) | <0.01 |
| Anemia | 128 (2.4) | 367 (4.1) | 253 (6.5) | <0.01 |
| Major bleeding | 528 (9.9) | 1 868 (20.8) | 1 032 (26.4) | <0.01 |
| Gastrointestinal | 160 (3.0) | 430 (4.8) | 298 (7.6) | <0.01 |
| Intracranial | 52 (1.0) | 268 (3.0) | 132 (3.4) | <0.01 |
| Other | 358 (6.7) | 1 347 (15.0) | 711 (18.2) | <0.01 |
| Renal failure | 54 (1.0) | 212 (2.4) | 77 (2.0) | <0.01 |
| Excessive alcohol use | 235 (4.4) | 292 (3.3) | 31 (0.8) | <0.01 |
| Fall | 1 015 (19) | 1 881 (21.0) | 1 402 (35.9) | <0.01 |
| Liver disease | 43 (0.8) | 104 (1.2) | 21 (0.5) | <0.01 |
| Cancer | 228 (4.3) | 1 305 (14.6) | 612 (15.7) | <0.01 |
| Cancer in GI-tract | 29 (0.5) | 235 (2.6) | 123 (3.2) | <0.01 |

^1^DVT/PE not classified as first or secondary excluded

^2^Percutanous coronary intervention

^3^Chronic obstructive pulmonary disease
