# Supplementary material for: Bleeding risk in patients with venous thromboembolic events treated with new oral anticoagulants
Source: J Thromb Thrombolysis. 2020 Nov 2;52(1):315–23. doi: 10.1007/s11239-020-02319-w (PMC8282556; doi:10.1007/s11239-020-02319-w)
Supplement: Supplementary file 2 — Supplementary file2 (DOCX 17 kb) [file 11239_2020_2319_MOESM2_ESM.docx]

# Bleeding risk in patients with venous thromboembolic events treated with new oral anticoagulants

*Authors*: Niklas Wallvik^1,3^, Henrik Renlund^2^, Anders Själander^1^

^1^ Umeå University, Department of Public Health and Clinical medicine, 981 87, Umeå, Sweden

^2^ Uppsala Clinical Research Center, Uppsala University, Uppsala, Sweden

^3^ Corresponding author. E-mail: niklas.wallvik@gmail.com

**Online Resource 2. Univariate and multivariate Cox regression analysis with major bleeding as endpoint.**

|  | Univariate | | Multivariate | |
| --- | --- | --- | --- | --- |
|  | HR (95% CI) | *p*-value | HR (95% CI) | *p*-value |
| Age, *normalized* (>1SD) | 1.56 (1.44-1.69) | <0.01 | 1.38 (1.27-1.50) | **<0.01** |
| Sex | 0.83 (0.73-0.95) | <0.01 | 0.96 (0.84-1.10) | 0.59 |
| First DVT/LE^1^ | 1.10 (1.94-1.31) | 0.22 | 0.90 (0.75-1.08) | 0.25 |
| Prior warfarin treatment | 0.79 (0.69-0.91) | <0.01 | 0.67 (0.58-0.78) | **<0.01** |
| Hypertension | 1.54 (1.27-1.87) | <0.01 | 1.17 (0.96-1.42) | 0.12 |
| Myocardial infarction | 1.44 (1.14-1.82) | <0.01 | 0.93 (0.62-1.39) | 0.74 |
| PCI^2^ | 1.61 (0.89-2.92) | 0.16 | 1.13 (0.61-2.10) | 0.70 |
| Atrial fibrillation | 1.65 (1.35-2.00) | <0.01 | 1.19 (0.97-1.46) | 0.10 |
| Heart Failure | 1.88 (1.49-2.36) | <0.01 | 1.28 (1.00-1.63) | 0.05 |
| Transient ischemic attack (TIA) | 2.00 (1.54-2.63) | <0.01 | 1.33 (1.01-1.76) | **0.04** |
| Stroke | 1.81 (1.47-2.22) | <0.01 | 1.28 (1.03-1.58) | **0.03** |
| Vascular disease | 1.48 (1.22-1.80) | <0.01 | 1.05 (0.75-1.48) | 0.76 |
| Diabetes | 1.08 (0.81-1.43) | 0.60 | 0.81 (0.61-1.07) | 0.14 |
| COPD^3^ | 1.51 (1.24-1.83) | <0.01 | 1.28 (1.04-1.60) | **0.02** |
| Dementia | 0.85 (0.47-1.54) | 0.59 | 0.58 (0.32-1.05) | 0.07 |
| Anemia | 1.69 (1.30-2.18) | <0.01 | 1.17 (0.89-1.53) | 0.26 |
| Major bleeding | 2.50 (2.19-2.86) | <0.01 | 1.58 (1.09-2.30) | **0.02** |
| GI bleeding | 2.65 (2.18-3.23) | <0.01 | 1.54 (1.11-2.13) | **0.01** |
| Intracranial bleeding | 2.12 (1.6-2.8) | <0.01 | 1.26 (0.85-1.87) | 0.25 |
| Other bleeding | 2.13 (1.84-2.47) | <0.01 | 1.19 (0.84-1.67) | 0.33 |
| Renal failure | 1.78 (1.21-2.59) | <0.01 | 1.27 (0.86-1.86) | 0.23 |
| Excessive alcohol use | 1.37 (1.00-1.88) | 0.051 | 1.36 (0.98-1.89) | 0.06 |
| Fall | 1.42 (1.23-1.64) | <0.01 | 1.13 (0.98-1.31) | 0.10 |
| Liver disease | 1.51 (0.87-2.61) | 0.14 | 1.29 (0.74-2.25) | 0.36 |
| Cancer | 1.46 (1.23-1.74) | <0.01 | 1.16 (0.96-1.39) | 0.18 |
| Cancer in GI-tract | 1.39 (0.95-2.03) | 0.09 | 0.94 (0.63-1.40) | 0.76 |
| ^1^DVT/PE not classified as first or secondary excluded ^2^Percutanous coronary intervention  ^3^Chronic obstructive pulmonary disease | | |  |  |
